# Neuroinflammatory alterations in trait anxiety: modulatory effects of minocycline

**DOI:** 10.1038/s41398-020-00942-y

**Published:** 2020-07-30

**Authors:** Sinead Rooney, Anupam Sah, Michael S. Unger, Maria Kharitonova, Simone B. Sartori, Christoph Schwarzer, Ludwig Aigner, Helmut Kettenmann, Susanne A. Wolf, Nicolas Singewald

**Affiliations:** 1 Department of Pharmacology and Toxicology, Institute of Pharmacy and Center for Molecular Biosciences Innsbruck (CMBI), University of Innsbruck, Innsbruck, Austria; 2 Institute of Molecular Regenerative Medicine, Paracelsus Medical University, Salzburg, Austria; 3 Spinal Cord Injury and Tissue Regeneration Center Salzburg (SCI-TReCS), Paracelsus Medical University, Salzburg, Austria; 4 Department of Pharmacology, Medical University of Innsbruck, Innsbruck, Austria; 5 Department of Cellular Neurosciences, Max Delbrück Center for Molecular Medicine, Berlin, Germany; 6 Department of Ophthalmology, Charité Universitätsmedizin, Berlin, Germany

**Keywords:** Psychiatric disorders, Neuroscience

## Abstract

High trait anxiety is a substantial risk factor for developing anxiety disorders and depression. While neuroinflammation has been identified to contribute to stress-induced anxiety, little is known about potential dysregulation in the neuroinflammatory system of genetically determined pathological anxiety or high trait anxiety individuals. We report microglial alterations in various brain regions in a mouse model of high trait anxiety (HAB). In particular, the dentate gyrus (DG) of the hippocampus of HABs exhibited enhanced density and average cell area of Iba1+, and density of phagocytic (CD68+/Iba1+) microglia compared to normal anxiety (NAB) controls. Minocycline was used to assess the capacity of a putative microglia ‘inhibitor’ in modulating hyperanxiety behavior of HABs. Chronic oral minocycline indeed reduced HAB hyperanxiety, which was associated with significant decreases in Iba1+ and CD68+Iba1+ cell densities in the DG. Addressing causality, it was demonstrated that longer (10 days), but not shorter (5 days), periods of minocycline microinfusions locally into the DG of HAB reduced Iba-1+ cell density and attenuated hyperanxiety-related behavior, indicating that neuroinflammation in the DG is at least partially involved in the maintenance of pathological anxiety. The present data reveal evidence of disturbances in the microglial system of individuals with high trait anxiety. Minocycline attenuated HAB hyperanxiety, likely by modulation of microglial activity within the DG. Thus, the present data suggest that drugs with microglia-targeted anti-inflammatory properties could be promising as novel alternative or complimentary anxiolytic therapeutic approaches in specific subgroups of individuals genetically predisposed to hyperanxiety.

## Introduction

Anxiety disorders are the most prevalent mental illnesses affecting 284 million people worldwide in 2017^1^. The median age of onset for anxiety in various anxiety disorders (11 years) is comparably earlier than in other psychopathologies such as mood disorders (30 years)^2^. Genetic predisposition to high trait anxiety has been identified as a severe risk factor for anxiety disorders and/or depression in later life^3^. New treatment approaches for such pathologies are necessary^4^ to overcome problems such as treatment-resistance, high suicide risk, and treatment complications by comorbidities. However, this requires a better understanding of underlying pathophysiological mechanisms^5^. Neuroinflammation has recently been recognized as a potential mechanism contributing to the onset and/or maintenance of psychiatric disorders, as well as to resistance to current treatments^6–8^. Specifically, presence of high inflammation and associated dysregulated downstream pathways has been linked with treatment resistance to antidepressants^9^, which are currently used as first line treatment in many anxiety disorders. Immune-targeting interventions have thus been proposed as an alternative route in the treatment of psychiatric disorders^10^. Neuroinflammation in the CNS involves key factors, such as microglial migration and activation, and can exert beneficial or detrimental consequences within an organism^11^.

The vast majority of clinical data in support of immune dysregulation contributing to the pathophysiology of anxiety-related disorders are restricted to those investigating peripheral levels of cytokines, in panic disorder^12,13^, post-traumatic stress disorder (PTSD)^14^, obsessive-compulsive disorder (OCD)^15,16^, and generalized anxiety disorder (GAD)^13,17–20^, with little attention given to social anxiety thus far. Pro-inflammatory or “activated” microglia secrete such cytokines and can phagocytose neural progenitor cells or parts of neurons (e.g., synaptic pruning), which shapes neuron circuits and can impact neuronal connectivity^21,22^. Interestingly, brain connectivity has been shown to be disturbed in early trait anxiety^23^. Recent positron-emission tomography (PET) methods of imaging translocator protein (TSPO) density as a marker, positively correlates microglial activation in brain regions, such as the hippocampus or dorsolateral prefrontal cortex, with scores of state anxiety and apathy^24^, although some have disputed TSPO as a specific marker for microglia^25^. In line with such observations in humans, alterations in microglia density or activation are reported in various animal models of stress-induced anxiety or depression, using e.g.: using unpredictable stress^26^ or repeated social defeat^27^ as stressors. Such inflammatory alterations are observed in key brain regions of the anxiety circuitry, the most consistently reported region being the hippocampus. Specifically, increased microglia density and/or coverage in the hippocampus by various stressor has been observed in rodents together with enhanced state anxiety^26,28–37^. Minocycline is a lipophilic broad-spectrum antibiotic drug with demonstrated anti-inflammatory properties, as well as being a reputed microglia activation ‘inhibitor’, and has been shown to significantly ameliorate stress-induced state anxiety in rodent models^31,32,38–40^. Thus far, there is a lack of studies investigating brain region-specific inflammatory changes in high trait anxiety, in the absence of specific stressors or immune challenge. Thus, detailed knowledge on how genetic predisposition to enhanced anxiety modulates the neuroinflammatory system is lacking.

The current study, therefore, investigated evidence of dysregulated neuroinflammation in the high anxiety (HAB) mouse model, which exhibits high trait anxiety compared to normal anxiety (NAB) controls. This phenotype is the result of a selective breeding approach of CD-1 mice, according to anxiety scores assessed in the elevated plus maze test^41,42^. Brain regions important in the neurocircuitry of anxiety include the hippocampus, amygdala, hypothalamus, and prefrontal cortex^43^, and HAB mice show altered activity processing in such anxiety-relevant networks and brain areas^44,45^. Interestingly, similar brain areas are likely candidate regions to show links between inflammatory and brain activity alterations, including the hippocampus, amygdala, and prefrontal cortex (e.g., refs. ^32,46^). The main aims of this study were to investigate whether mice with high trait anxiety display alterations in the brain microglia system as compared to normal anxiety (NAB) control mice, and whether the aberrant anxiety behavior can be modulated by a drug possessing anti-inflammatory properties by targeting microglia, minocycline.

## Materials and methods

For detailed information, see Supplementary Materials and Methods.

### Animals

Male HAB and NAB mice were selectively inbred for their specific anxiety-related behavior at the Department of Pharmacology, Innsbruck Medical University, Innsbruck (Austria), as described previously^47^. HABs and NABs (11-22w) had access to food pellets and water ad libitum, and were group-housed in individually ventilated cages under standard laboratory conditions (12:12 light/dark cycle with lights on at 07:00 h, 22 ± 2 °C, 45–60% humidity). All experiments were approved by the Austrian Animal Experimentation Ethics Board (Bundesministerium für Wissenschaft Forschung und Wirtschaft, Kommission für Tierversuchsangelegenheiten) and were in compliance with international laws and policies.

### Minocycline treatment

Minocycline dosages for systemic and local administration were chosen according to previous studies showing effects on microglia and behavior^26,31^. Mice received an average oral minocycline (Sigma-Aldrich) dosage of 40 mg/kg/day for 28 d. For local microinjections, minocycline (20 µg/μl) was infused bilaterally into the DG once daily, for 5 d (shorter period) or 11 d (longer period).

### Stereotaxic surgery and microinjections

HAB mice were placed in a stereotaxic frame (David Kopf Instruments) under 2% isoflurane anesthesia. Guide cannulas (25 gauge, 8 mm in length) were implanted 1 mm above the left and right DG (AP: −2.18 mm, ML: ±1.40 mm, DV: −1.2 mm from Bregma). Following surgery, mice were single-housed and received buprenorphine (0.5 mg/kg s.c.) and meloxicam (0.5 mg/kg p.o. via drinking water) for analgesic care for up to 3 d, and were allowed to recover for 7 d. Either saline or minocycline solution (0.25 µl/hemisphere) were infused bilaterally at a speed of 0.1 µl/min. The histological verification of the localization of the microinfusion probes revealed one animal with misplaced cannulae that was subsequently removed from the further behavioral or microglial analysis.

### Behavioral testings

Levels of anxiety-related behavior of mice were assessed in the light/dark (LD) test, open field test, and the elevated plus maze (EPM) test according to established and previously used protocols^47,48^.

### Immunohistochemistry

Mice were sacrificed 2 h following the LD test. Coronal brain sections were incubated with primary antibodies, goat anti-Iba1 (1:500 Abcam, #ab107159) and rabbit anti-CD68 (1:300 Abcam, #ab125212) or rabbit anti-TMEM119 (1:300 Abcam, #ab209064) followed by incubation with respective fluorescent-labeled secondary antibodies using established immunohistochemistry protocols^47^.

### Immunofluorescence microscopy

In one section per mouse, images of both the left and right DG of the hippocampus (Interaural 1.98 mm, Bregma −1.82 mm), representative area consisting of polymorphic, hilus and granular cell layers, were taken using a fluorescent microscope (Olympus, Austria), applying a ×4 objective to locate specific brain structures and a ×20 objective for quantitative analyses. Additional images were taken of the basolateral amygdala (BLA), nucleus accumbens (NAcc), medial prefrontal cortex (mPFC), cingulate cortex, and paraventricular nucleus (PVN) of the hypothalamus. For oral minocycline experiment, both the left and right DG (−1.82 mm from Bregma) was assessed. For intra-DG minocycline experiment, Iba-1+ cells were counted at the level of (−2.80 mm from Bregma) due to significant tissue tear at the level of −1.82 mm that was observed in almost all animals. Quantification of immunopositive-cells was assisted by an image analysis system (cellSens Dimension; Olympus).

### Statistical analysis

Data analyses were performed with GraphPad Prism 8.0 software (GraphPad Software Inc., USA), following the exclusion of outliers identified by Grubb’s test. Data were analyzed by unpaired Student’s *t*-test (two-tailed). Correlational analysis was evaluated by Pearson’s co-efficiency analysis. Significance was set at *p* < 0.05, and data are presented as means ± standard error of the mean (S.E.M.).

## Results

### Microglia alterations in the brains of high trait anxiety mice

**Fig. 1:**
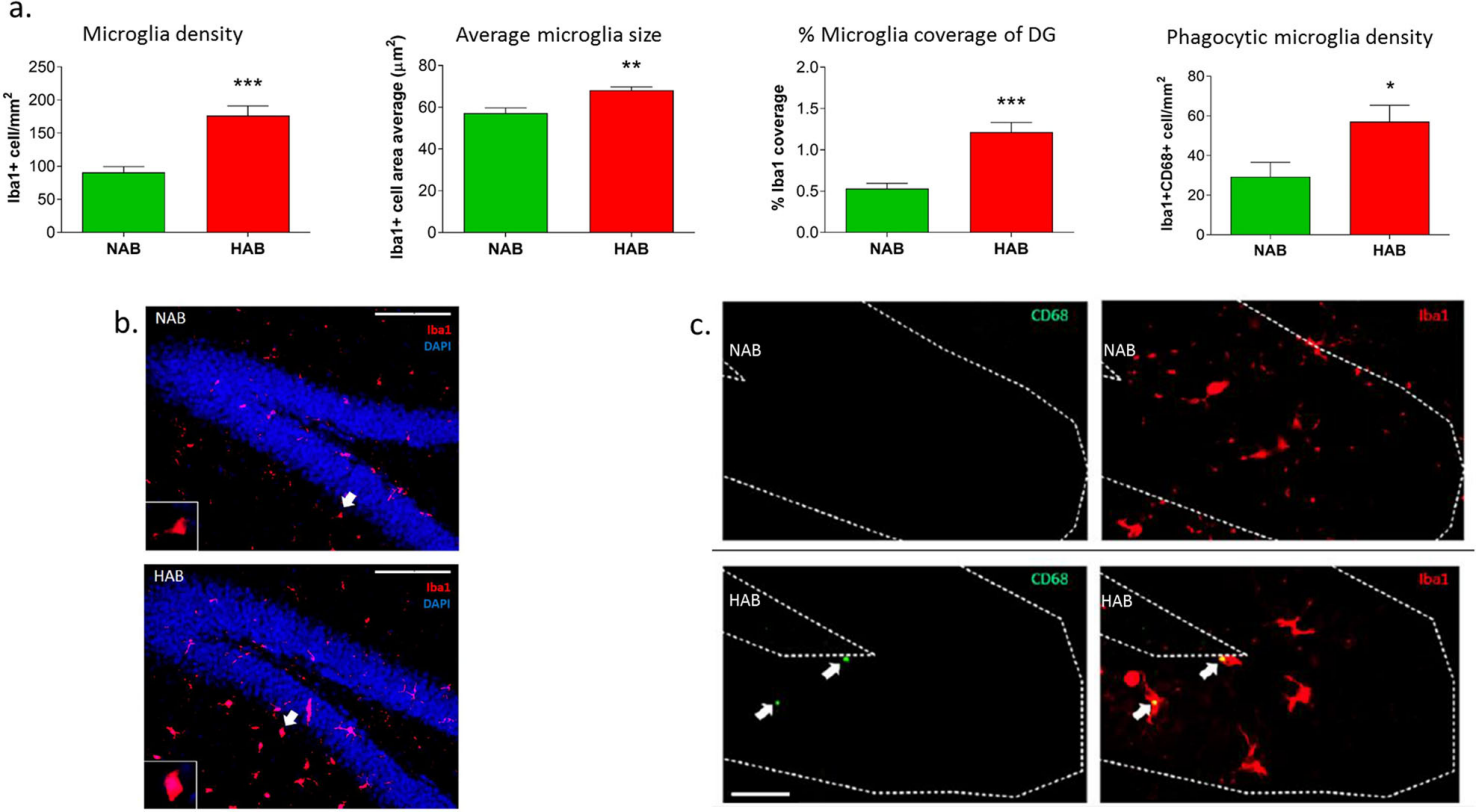
Microglial alterations in the dentate gyrus (DG) of HAB mice. Hyperanxious HAB mice show significantly enhanced Iba1^+^ cell density, Iba1^+^ cell area average, and percentage of coverage by Iba1+ cells of the dorsal DG, compared to NAB controls (**a**). Representative images of Iba1 (red) and DAPI (blue) staining in the DG of HAB and NAB mice, inset is microglia size example (**b**) scale bar 100μm. Iba1^+^ CD68^+^ cell density is enhanced in the granular cell layer of the DG in HAB (**a**). Representative images of Iba1 (red) and CD68 (green) staining in the DG of HAB and NAB mice (**c**), scale bar 50 μm. Data are presented as mean ± SEM. *n* = 7–11. *p < 0.05, **p <0.01, ***p < 0.001 vs. NAB (Student’s *t* - test).

In hyperanxious HAB mice and normal anxiety NAB controls, Iba1+ microglia were imaged and visualized in various anxiety-related brain regions. Among all regions analyzed, the DG, the neurogenic niche of the hippocampus, showed the most robust Iba1 alterations (Fig. S1). Our previous studies have shown altered neuronal activity and reduced neurogenesis associated with anxiety in the DG of HAB mice and rats^45,47^, thus compounding the DG as the main candidate region of interest. Image J-assisted analysis revealed that the density (*p* < 0.001, *t*_(16)_ = 4.311) and average cell area (*p* < 0.01, *t*_(11)_ = 3.695) of Iba1+ microglia was significantly higher in the dorsal DG (dDG) of HAB mice, compared to NAB mice (Fig. 1a, b). Using Image J-generated total area (µm^2^) of Iba1-positive staining, this was divided by the total area of the image (µm^2^) and expressed as a percentage (% Iba1 coverage), which can be an index used to account for simultaneous alterations in cell density and morphology^49^. The percentage of Iba1 coverage was enhanced in the dDG of HAB (*p* < 0.001, *t*_(16)_ = 4.288; Fig. 1a). In the ventral DG, the density (*p* < 0.05, *t*_(16)_ = 2.647), average cell area (*p* < 0.05, *t*_(16)_ = 2.382) and % coverage (*p* < 0.05, *t*_(16)_ = 2.427) of Iba1+ microglia was also significantly increased in HAB compared to NAB (Figs. S1 and S2a). In regards to additional brain regions, there was an augmentation of average Iba1+ cell area (*p* < 0.01, *t*_(11)_ = 3.304) and % Iba1 coverage (*p* < 0.05, *t*_(11)_ = 2.395) observed in the medial prefrontal cortex, but changes in density did not reach statistical significance (*p* > 0.05, *t*_(11)_ = 1.795) (Fig. S2b). There were no significant differences in other brain regions investigated including the cingulate cortex, BLA, PVN, and NAcc (Fig. S1).

Since Iba1 is a constitutive marker for myeloid cells, we used TMEM119 as an additional marker for the discrimination of resident microglia from potentially infiltrating blood-derived macrophages^50^. Our findings revealed that the vast majority of Iba1+ cells were positive for TMEM119 (98.91 ± 0.24%) in the DG of HAB mice, indicating the vast majority of the enhanced Iba1+ cells represents a true microglia population (Fig. S3). Specific cellular markers expressed on microglia are important indicators of microglial activity in the central nervous system (CNS), thus we next aimed to determine the expression levels of the phagocytosis/antigen-presentation marker CD68^51,52^ in the DG. There was an increased density of co-labeled Iba1+CD68+ cells in the granular cell layer of HAB, compared to NAB (*p* < 0.05, *t*_(16)_ = 2.330; Fig. 1a, c), indicating a potential increase in microglial phagocytic activation in this region in HAB compared to NAB mice.

### Anxiety-like behavior of HAB and NAB mice was correlated with microglia alterations in the DG

**Fig. 2:**
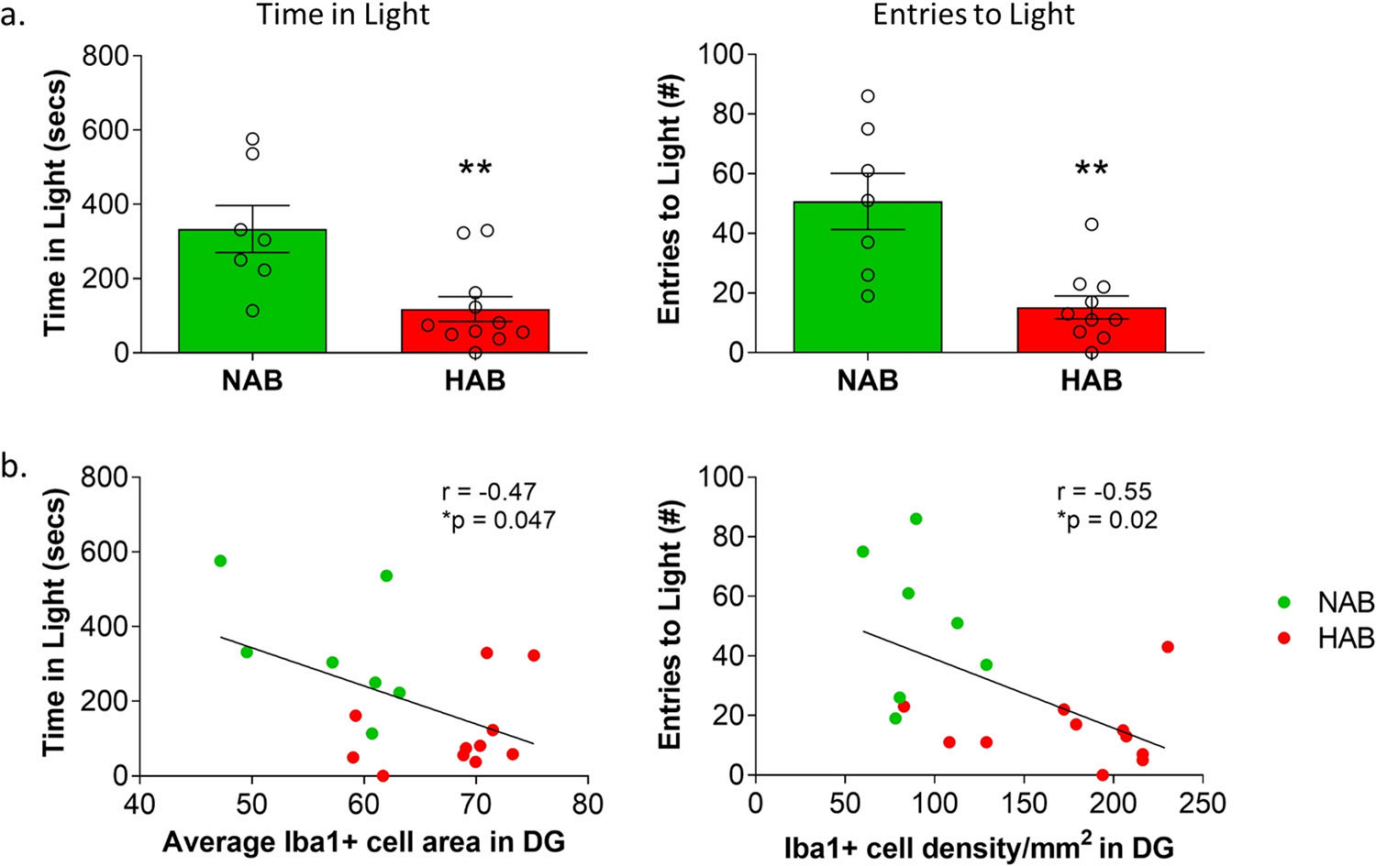
Anxiety behavior is correlated with microglia alterations in the dentate gyrus (DG) of HAB mice. HAB mice display hyperanxious behavior in the light/dark test, whereby HAB mice spent significantly less time in the light arena and entered the light arena less often, compared to NAB (**a**). In HAB and NAB, Iba1^+^ microglia average cell area and Iba1^+^ cell density in the DG was negatively correlated with behavioral scores in the light/dark test; time spent in and entries to the light arena, respectively (**b**). *p < 0.05, Pearson’s correlation, *r* and *p* values indicated in the plots. *n* = 7–11.

Behavioral testing in the LD test confirmed an enhanced anxiety-related behavior in HABs, compared to NAB controls (Fig. 2a), as HAB mice spent less time in the brightly lit compartment of the testing arena (*p* < 0.01, *t*_(15)_ = 3.146), and also entered the light arena less often (*p* < 0.01, *t*_(8)_ = 3.484), as demonstrated previously^47,53^. To gain evidence linking behavior and neuroinflammatory parameters, we asked whether alterations in hippocampal microglia are associated with symptom severity of trait anxiety in HAB and NAB groups. Indeed, the average microglia area size in the DG was negatively correlated with time spent in the light (*p* < 0.05, *r* = −0.47), and microglia density in the DG was negatively correlated with entries to the light (*p* < 0.05, *r* = −0.55) (Fig. 2b), indicating that enhanced microglial density or size within the DG correlates with enhanced anxiety in the LD test.

### Attenuation of hippocampal microglia activity and anxiety-related behavior in HAB mice by chronic oral minocycline treatment

**Fig. 3:**
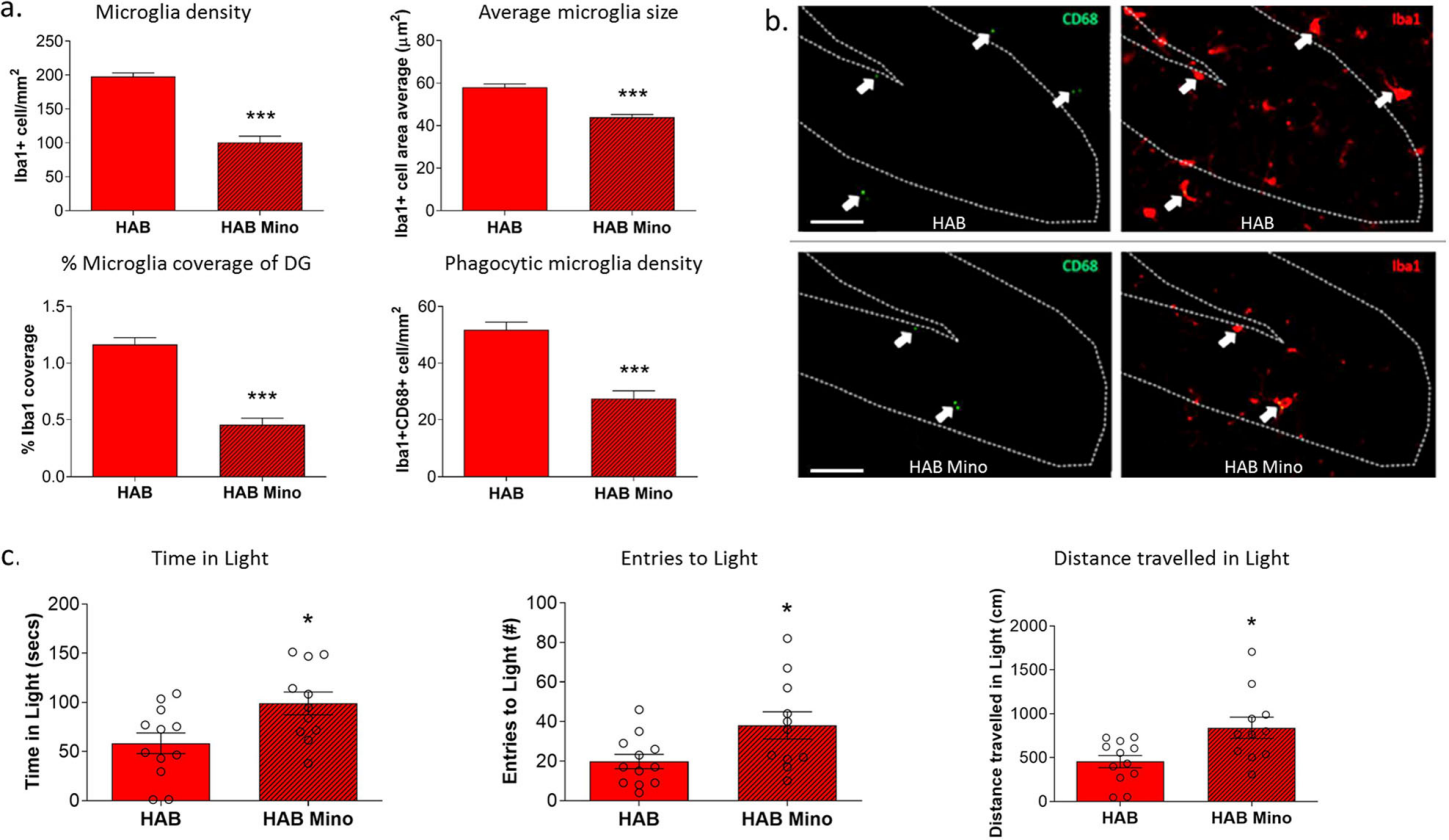
The impact of chronic oral minocycline treatment on microglia in the dentate gyrus (DG) and anxiety-related behavior of HAB mice. HAB mice treated with oral minocycline (Mino) showed reduced Iba1^+^ cell density, Iba1^+^ cell area average, percentage of coverage by Iba1+ cells of the DG, and CD68^+^Iba1^+^ microglia density, compared to untreated HAB (**a**). Representative images of Iba1 (red) and CD68 (green) staining in the DG of HAB and HAB Mino groups (**b**), scale bar 50 μm. HAB mice show reduced anxiety behavior in the light/dark test following minocycline treatment, whereby HAB Mino group spent significantly more time in, more entries to, and more distance traveled in, the light arena compared to untreated HAB (**c**). Data are presented as mean ± SEM. *n* = 10–13. *p < 0.05, **p < 0.01, ***p < 0.001 (Student’s *t*-test).

Minocycline was administered in order to substantiate the association between neuroinflammation and high trait anxiety, and to gain information whether the observed elevation in microglia activity in the DG contributes to or represents an expression of insufficient counter-regulation to pathological hyperanxiety. In HAB mice, chronic oral minocycline treatment reduced the enhanced Iba1+ microglia density (*p* < 0.001, *t*_(18)_ = 8.741), average cell area size (*p* < 0.001, *t*_(18)_ = 6.840) and percentage of coverage of the dDG (*p* < 0.001, *t*_(18)_ = 8.486) compared to vehicle treatment (Fig. 3a). Additionally, there was an attenuation of co-labeled Iba1+CD68+ microglia density in the granular cell layer of the dDG (*p* < 0.001, *t*_(19)_ = 6.266; Fig. 3a, b) in minocycline- vs. vehicle-treated HAB mice. Therefore, we next asked whether minocycline-induced attenuation of microglia was associated with a reduction in hyperanxiety. Indeed, we found that minocycline reduced enhanced anxiety-like behavior in HABs, as in the light–dark test, minocycline-treated HAB mice displayed an increased amount of time spent in (*p* < 0.05, *t*_(21)_ = 2.612), number of entries to (*p* < 0.05, *t*_(21)_ = 2.415), and distance traveled in (*p* < 0.05, *t*_(21)_ = 2.815), the light arena compared to vehicle-treated HABs (Fig. 3c).

### Longer, but not shorter, periods of intra-DG minocycline treatment attenuated hyperanxiety of HAB mice accompanied by reduced microglial density in the DG

**Fig. 4:**
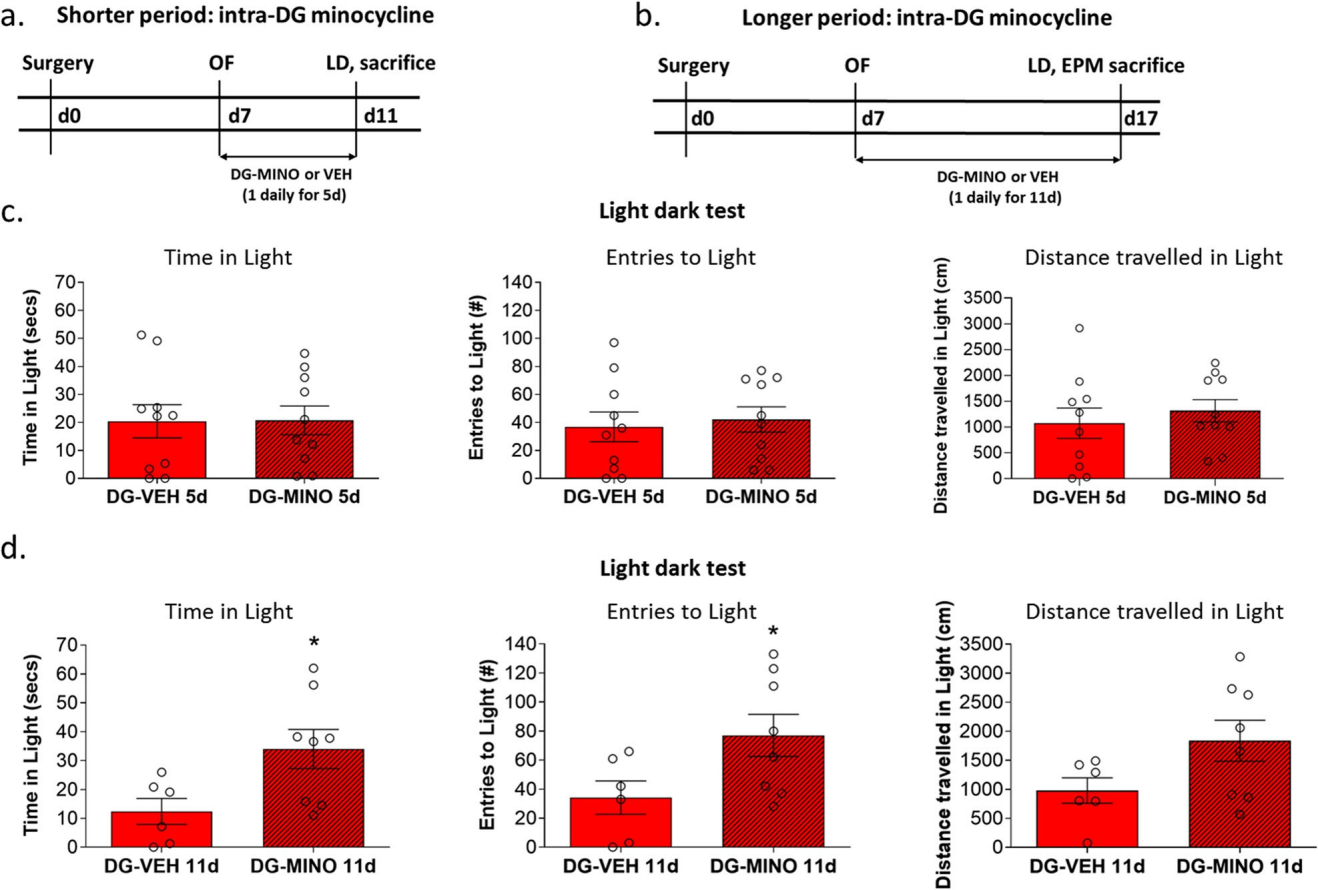
Impact of local application of into the dentate gyrus (DG) on anxiety-like behavior of HAB mice. Experimental timelines, shorter period (5 d) (**a**) and longer periods (11 d) (**b**) of minocycline infusions. Following 5 days of intra-DG minocycline or saline daily infusions, HAB groups showed no significant differences in the light/dark test (**c**). Following 10 days of intra-DG minocycline daily infusions, HAB displayed increased time spent in, entries to, and distance traveled in, the light arena of the light/dark test (**d**), compared to intra-DG saline HAB. Data are presented as mean ± SEM. *n* = 7–11, *p < 0.05, **p < 0.01, ***p < 0.001 (Student’s *t*-test).

**Fig. 5:**
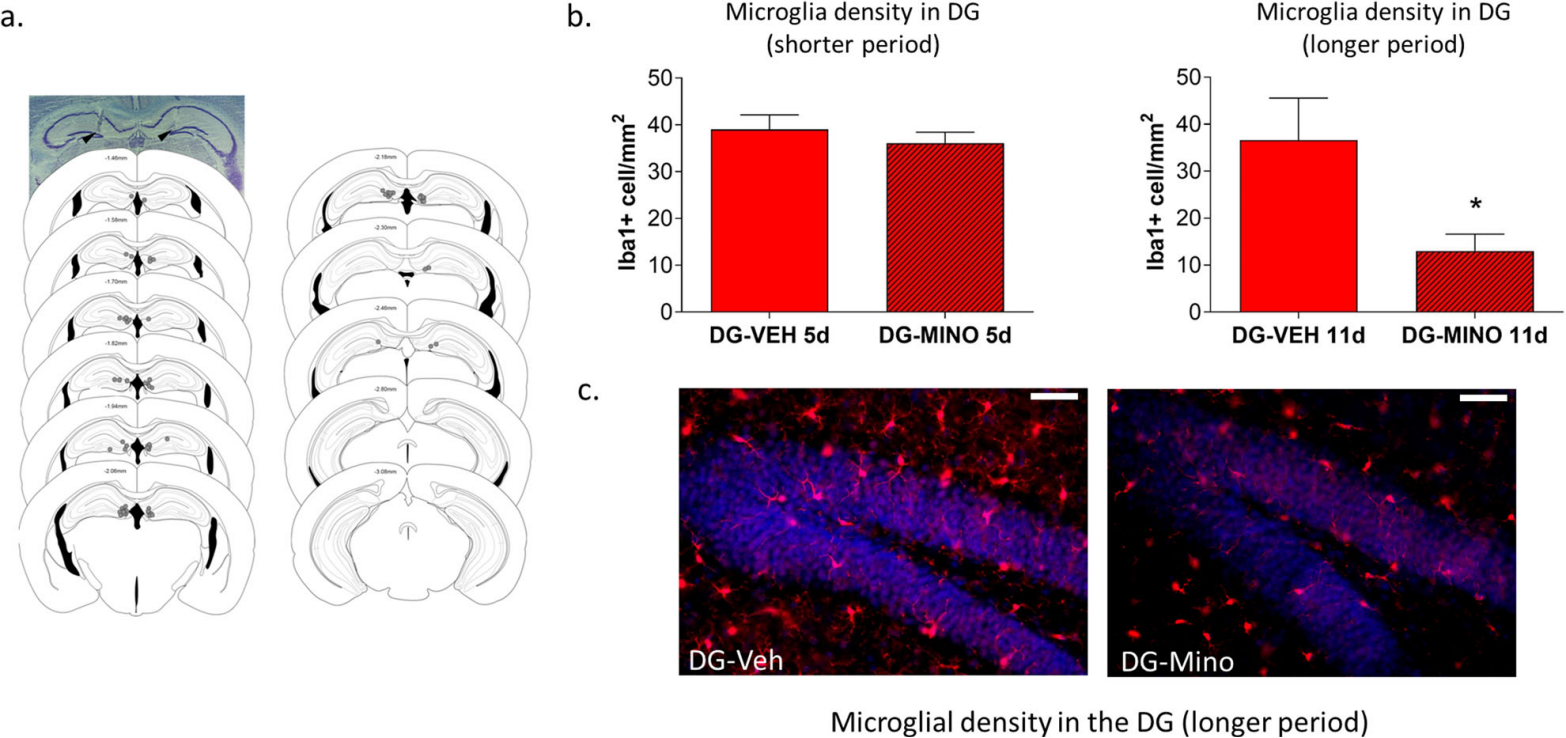
Impact of local application of minocycline into the dentate gyrus (DG) on Iba-1 density in HAB mice. Schematic illustration of the sites of DG hits (a). Following 5 days of intra-DG minocycline or saline daily infusions, HAB groups showed no significant differences in the Iba-1 density (**b**). Following 10 days of intra-DG minocycline daily infusions, HAB showed reduced Iba-1 density (**b**). Representative images showing Iba-1+ cells in the DG following long-term intra-DG vehicle/ minocycline administration (**c**). Data are presented as mean ± SEM. *n* = 7–11, *p < 0.05 (Student’s *t*-test).

In order to investigate a direct link between trait anxiety behavior and microglial activity in the DG, minocycline was microinjected bilaterally into the DG of HAB for a shorter (5 days, Fig. 4a) or longer period (11 days, Fig. 4b). To test for acute effects, animals were subjected to the open field test at 2 h following the first microinjection. This test was chosen here instead of the LD test in order to avoid habituation effects upon second test exposure along with a possible loss of sensitivity in detecting anxiolytic effects^54^. N12o significant differences were found in both experiments (*p* > 0.05, Fig. S4), thus indicating no acute effect of minocycline on anxiety-related behavior. After repeated administration, mice of all groups were subjected to the LD test at 2 h following the last microinjection. Mice treated for 5 days showed no significant differences in time spent in entries to, and distance traveled in, the light arena, compared to the vehicle group (*p* > 0.05) (Fig. 4c). This lack of effect on anxiety-related behavior was paralleled with no changes in the Iba-1+ cell density n the DG (Fig. 5b).

In contrast, longer (10 days) intra-DG minocycline-treated HAB displayed significantly increased time spent in (*p* < 0.05, *t*_(12)_ = −2.470), entries to (*p* < 0.05, *t*_(12)_ = −2.186), and distance traveled in (*p* = 0.08, *t*_(12)_ = −1,889), the light arena (Fig. 4d) compared to controls. On the next day the anxiolytic-like effect of long-term intra-DG minocycline was explored in an additional test to assess anxiety-related behavior, the elevated plus maze. Again, we obtained evidence of reduced anxiety-related behavior of long-term intra-DG minocycline-treated HAB as indicated by a significant increase in percentage of entries to the open arm of the EPM (*p* < 0.05, *t*_(11)_ = 2.524) (Fig. S5), thereby validating the results of the LD test. Furthermore, in association with successful anxiolysis, longer periods of intra-DG minocycline treatment exerted a significant reduction in Iba-1+ cell density in the DG of HABs (Fig. 5b, c), compared to vehicle-treated HABs.

## Discussion

The current results show that HAB mice, possessing a genetic predisposition to high trait anxiety, display clear signs of a central immune dysregulation as indicated by an increased density of microglial phagocytic activity in the DG. Minocycline, a tetracycline antibiotic demonstrating significant anti-inflammatory properties^55^, partially improved the revealed microglia dysregulation in the CNS, upon systemic administration. These minocycline-induced effects on microglia were associated with clearly attenuated hyperanxious behavior in HABs. Furthermore, modulating microglia activity locally in the DG, via long-term minocycline microinjection, was also sufficient to attenuate hyperanxiety in HAB. Together, the current findings suggest that central inflammatory disturbances are evident in individuals with high trait anxiety, even in the absence of known triggers of inflammation such as chronic stress^56,57^. Furthermore, inflammation-targeting approaches, such as inhibitors of microglia ‘activation’, could serve as alternative or complementary treatment approaches in patient sub-groups presenting with genetically determined hyperanxiety.

The role of neuroinflammation in trait anxiety has remained virtually unexplored, with the exception of one study comparing varying levels of anxiety in mice, however in unrelated mouse strains that differ not only in anxiety, but also in a variety of other behaviors^58^. Although this study did not investigate differences in resident microglia population, it showed that an increased ratio of ‘M1 (pro-inflammatory)’ to ‘M2 (anti-inflammatory)’ microglia was present in whole brain homogenates of a high anxiety DBA/2 J strain. Here we compare microglia across various brain regions of innate high- and normal-anxiety mice selectively bred from the same genetic CD-1 background, thus reducing between-strain differences. Our findings reveal that even in the absence of immune challenge or specific stress paradigms, HAB mice exhibiting high trait anxiety showed increased microglia density, cell size and coverage specifically in the DG of the hippocampus, a brain region implicated in various functions including anxiety, learning and memory^43,59^, indicating a contributing role for neuroinflammation in the disruption of these processes.

Enhanced microglia density and activity in the hippocampus of HABs is in line with evidence from a human PET study in which microglial activation (as measured by TSPO binding capacity) in the hippocampus was positively correlated with anxiety scores^24^. Furthermore, anxiety- and depression-like behaviors induced by chronic mild stress^57^ or postnatal stress^60^ are associated with increased microglial ‘activation’ in the hippocampus, and acute unpredictable stress increases microglia density in the DG of mice^26^. Microglia are regulators of neurogenic activity^61^ and suppressed neurogenesis following acute stress has been shown to be accompanied by increased presence of microglia in the DG^30^. In that respect it is interesting to note that neurogenesis and functional integration of newly born neurons are impaired in the DG of HAB^47^ and neuronal activation in the DG was found to be blunted (c-Fos expression in response to different challenges)^45,47^. These observations together with the current data indicate a link between reduced hippocampal neurogenesis/connectivity and enhanced microglial density/activation in high trait anxiety HAB, which will be addressed in future studies.

Relatedly, microglial phagocytic activity seems to be enhanced in the DG of hyperanxious HAB as indicated by an increased CD68^+^ microglia density, compared to NAB controls. Along this line, CD68 immunoreactivity has been shown to be enhanced in the hippocampus of chronic unpredictable mild stress-exposed rats displaying increased anxiety behavior^28^, and also in the DG of stress animal models (early life stress;^29^ forced swim stress^30^). Thus the involvement of enhanced hippocampal CD68 expression, and thereby microglial phagocytic activity (CD68+Iba1+), previously demonstrated in state anxiety is now also supported in trait anxiety model. Taken together, these studies indicate that microglia state and function contribute to the maintenance of both state and trait anxiety.

We found that the enhanced DG microglial density in HAB mice can be normalized with chronic systemic minocycline administration, which was in association with significant anxiolysis in these hyperanxious mice. While the present study indicates that chronic minocycline (28 d) can attenuate trait anxiety in HAB mice in the light/dark test, a shorter minocycline treatment protocol (17 d) did not attenuate hyperanxiety in HAB rats^62^, assessed in the light/dark test or EPM. This might be caused by the difference in treatment duration, but as well could reflect interspecies differences. In line with the latter, HAB mice have been shown to be SSRI-insensitive^63^, while HAB rats respond to some, but not all, SSRIs^62,64^. Furthermore, rat and mouse HAB lines were generated independently by selective breeding, therefore, it is likely, that HAB mice/rats may reflect differing aspects of anxiety disorders. In support of this idea, pilot studies in humans have shown that minocycline as monotherapy or in combination with antidepressants/antipsychotics leads to moderate improvement in anxiety scores of patients with treatment-resistant depression^65^, obsessive compulsive disorder^66^, schizophrenia^67^ or Fragile-X syndrome^68^. Along these lines, investigation of the effectiveness of minocycline in the treatment of chemotherapy-induced anxiety and depression in breast cancer patients is currently registered (NCT02203552). Also recently, another clinical trial has been initiated to assess the effect of minocycline as an adjunctive treatment (to standard antidepressants) for treatment-resistant depression and comorbid GAD^69^.

The minocycline-induced attenuation of hyperanxiety in HABs was also associated with a significant reduction of the enhanced CD68^+^ microglia population in the DG, which indicates that minocycline exerts its anxiolytic effects potentially by reducing microglial phagocytic activity in this region. Along these lines, the administration of coenzyme Q10, an antioxidant and anti-inflammatory compound, was shown to decrease the stress-induced increase in hippocampal CD68 immunoreactivity in a dose-dependent manner, in association with a decrease of anxiety behavior^28^. Together, such drug studies support an association between reduced brain CD68 expression and attenuated anxiety.

Interestingly, microglial differences between HAB and NAB were heterogenous in different brain areas. While such differences were minor or missing in brain areas such as nucleus accumbens, PVN or BLA, we found increased Iba1+ microglia cell area size, coverage and density also in the mPFC (medial prefrontal cortex) of HAB mice. In contrast, a recent study in rats showed lower Iba-1+ cell density within the mPFC of HAB vs NAB^62^. A reason for this discrepancy could be that Schmidtner and colleagues^62^ exposed HAB rats to multiple challenges involving LD test, EPM test, pre-swim session, forced swim test, while HAB mice in the current study were exposed to only one challenge (LD test). Furthermore, the mPFC is a heterogeneous cortical structure composed of different subregions (including the prelimbic and infralimbic cortex) which are differentially involved in anxiety-like behavior^70^. Furthermore, microglial cells are distinctly expressed in a layer-specific manner within these subregions which are again differentially regulated by stressors^71^. Therefore, regional differences as well as single/multiple stressors could account for the discrepancy in microglial density in both the studies. Nevertheless, both these studies across two differing rodent species suggest that besides the DG, the mPFC represents an additional inflammatory focal point in the CNS of individuals predisposed to trait anxiety and it will be also interesting to see how anxiety can be modulated by interfering with the inflammatory system within the mPFC.

Evidence of a possible causal link between enhanced DG microglia density and trait anxiety behavior is provided in the current study by pharmacologically targeting local DG microglia (and possibly other CNS cells) with minocycline microinjection. This reduced DG Iba-1+ expression and hyperanxiety in HAB, as measured by the LD test. Notably, only when minocycline was able to reduce DG microglia in a longer 10 d (but not in a 5 d) treatment protocol, anxiolysis was observed, indicating that it is likely necessary to recruit mechanisms that require more chronic interaction, e.g.: enhancing neuroplasticity and/or neurogenesis in this brain area. However, the possibility that local infusion of minocycline to the DG could also exert effects on microglia and/or cytokines in other brain regions, as well as immune cells in the periphery cannot be fully excluded. Only few studies thus far have investigated the effects of local application of minocycline on anxiety or anxiety-related behaviors. Acute stress-induced anxiety in rats, induced by single prolonged stress, was shown to be attenuated by application of intra-hippocampal minocycline^31^. In general, such studies altering anxiety behavior by directly modulating microglia activity in a brain region-specific manner support the current findings, which suggest neuroinflammation in the DG of the hippocampus as a mechanism contributing to the maintenance of pathological anxiety.

In conclusion, this study describes brain region-specific microglial alterations in a mouse model of high trait anxiety. These alterations were sensitive to the microglia-modulating drug, minocycline. Specifically, chronic systemic or local intra-DG minocycline was sufficient to reduce microglial upregulation and hyperanxious behavior in this mouse model. Thus, treatments including drugs^72^ or also antibody approaches^73^ targeting microglia and/or additional inflammatory mechanisms represent potential strategies for a subgroup of individuals with high trait anxiety who are at a risk of developing neuropsychiatric disorders such as anxiety disorders or depression.

## Supplementary information

Supplementary Materials and Methods

Supplementary Fig S1

Supplementary Fig S2

Supplementary Fig S3

Supplementary Fig S4

Supplementary Fig S5

## References

[1] Number with a mental or neurodevelopmental disorder by type, World, 2017. https://ourworldindata.org/grapher/number-with-mental-and-neurodevelopmental-disorders-by-type (2017).

[2] Kessler RC (2005). Lifetime prevalence and age-of-onset distributions of DSM-IV disorders in the National Comorbidity Survey Replication. Arch. Gen. Psychiatry.

[3] Weger M, Sandi C (2018). High anxiety trait: a vulnerable phenotype for stress-induced depression. Neurosci. Biobehav. Rev..

[4] Sartori S. B. & Singewald N. Novel pharmacological targets in drug development for the treatment of anxiety and anxiety-related disorders. *Pharmacol. Ther.* **204**, 107402 (2019). 10.1016/j.pharmthera.2019.107402 31470029

[5] Craske MG (2017). Anxiety disorders. Nat. Rev. Dis. Prim..

[6] Goldsmith DR, Rapaport MH, Miller BJ (2016). A meta-analysis of blood cytokine network alterations in psychiatric patients: comparisons between schizophrenia, bipolar disorder and depression. Mol. Psychiatry.

[7] Michopoulos V, Powers A, Gillespie CF, Ressler KJ, Jovanovic T (2017). Inflammation in fear- and anxiety-based disorders: PTSD, GAD, and beyond. Neuropsychopharmacology.

[8] Yirmiya R, Rimmerman N, Reshef R (2015). Depression as a microglial disease. Trends Neurosci..

[9] Haroon E (2018). Antidepressant treatment resistance is associated with increased inflammatory markers in patients with major depressive disorder. Psychoneuroendocrinology.

[10] Radtke FA, Chapman G, Hall J, Syed YA (2017). Modulating neuroinflammation to treat neuropsychiatric disorders. Biomed. Res. Int.

[11] DiSabato DJ, Quan N, Godbout JP (2016). Neuroinflammation: the devil is in the details. J. neurochemistry.

[12] Petrowski K, Wichmann S, Kirschbaum C (2018). Stress-induced pro- and anti-inflammatory cytokine concentrations in panic disorder patients. Psychoneuroendocrinology.

[13] Zou Z (2020). Differences in cytokines between patients with generalised anxiety disorder and panic disorder. J. Psychosom. Res..

[14] Passos IC (2015). Inflammatory markers in post-traumatic stress disorder: a systematic review, meta-analysis, and meta-regression. Lancet Psychiatry.

[15] Fontenelle LF (2012). A cytokine study of adult patients with obsessive-compulsive disorder. Compr. Psychiatry.

[16] Rao NP (2015). Plasma cytokine abnormalities in drug-naive, comorbidity-free obsessive-compulsive disorder. Psychiatry Res.

[17] Hou R (2017). Peripheral inflammatory cytokines and immune balance in generalised anxiety disorder: case-controlled study. Brain, Behav., Immun..

[18] Khandaker GM, Zammit S, Lewis G, Jones PB (2016). Association between serum C-reactive protein and DSM-IV generalized anxiety disorder in adolescence: findings from the ALSPAC cohort. Neurobiol. Stress.

[19] Oglodek EA, Szota AM, Just MJ, Mos DM, Araszkiewicz A (2015). The MCP-1, CCL-5 and SDF-1 chemokines as pro-inflammatory markers in generalized anxiety disorder and personality disorders. Pharm. Rep..

[20] Tang Z (2018). Peripheral proinflammatory cytokines in Chinese patients with generalised anxiety disorder. J. Affect. Disord..

[21] Kettenmann H, Hanisch UK, Noda M, Verkhratsky A (2011). Physiology of microglia. Physiol. Rev..

[22] Kettenmann H, Kirchhoff F, Verkhratsky A (2013). Microglia: new roles for the synaptic stripper. Neuron.

[23] Kalin NH (2017). Mechanisms underlying the early risk to develop anxiety and depression: a translational approach. Eur. Neuropsychopharmacol..

[24] Hafizi S (2017). Imaging microglial activation in individuals at clinical high risk for psychosis: an in vivo PET Study with [(18)F]FEPPA. Neuropsychopharmacology.

[25] Notter T (2018). Translational evaluation of translocator protein as a marker of neuroinflammation in schizophrenia. Mol. Psychiatry.

[26] Kreisel T (2014). Dynamic microglial alterations underlie stress-induced depressive-like behavior and suppressed neurogenesis. Mol. Psychiatry.

[27] Wohleb ES, Delpech JC (2017). Dynamic cross-talk between microglia and peripheral monocytes underlies stress-induced neuroinflammation and behavioral consequences. Prog. Neuropsychopharmacol. Biol. Psychiatry.

[28] Abuelezz SA, Hendawy N, Magdy Y (2017). Targeting oxidative stress, cytokines and serotonin interactions via indoleamine 2, 3 dioxygenase by coenzyme Q10: ROLE IN SUPPRESSING DEPRESSIVE LIKE BEHAVIOR IN RAts. J. Neuroimmune Pharm..

[29] Hoeijmakers L (2017). Early-life stress lastingly alters the neuroinflammatory response to amyloid pathology in an Alzheimer’s disease mouse model. Brain, Behav., Immun..

[30] Llorens-Martin M, Jurado-Arjona J, Bolos M, Pallas-Bazarra N, Avila J (2016). Forced swimming sabotages the morphological and synaptic maturation of newborn granule neurons and triggers a unique pro-inflammatory milieu in the hippocampus. Brain, Behav. Immun..

[31] Sun R. et al. Hippocampal activation of microglia may underlie the shared neurobiology of comorbid posttraumatic stress disorder and chronic pain. *Mol. Pain* **12**, 1744806916679166 (2016). 10.1177/1744806916679166 PMC5117253 27852966

[32] Wang W (2018). Minocycline attenuates stress-induced behavioral changes via its anti-inflammatory effects in an animal model of post-traumatic stress disorder. Front. Psychiatry.

[33] Wohleb ES (2012). Peripheral innate immune challenge exaggerated microglia activation, increased the number of inflammatory CNS macrophages, and prolonged social withdrawal in socially defeated mice. Psychoneuroendocrinology.

[34] Wohleb ES (2011). beta-Adrenergic receptor antagonism prevents anxiety-like behavior and microglial reactivity induced by repeated social defeat. J. Neurosci..

[35] Wohleb ES (2014). Knockdown of interleukin-1 receptor type-1 on endothelial cells attenuated stress-induced neuroinflammation and prevented anxiety-like behavior. J. Neurosci..

[36] Zhang C (2019). Minocycline ameliorates depressive behaviors and neuro-immune dysfunction induced by chronic unpredictable mild stress in the rat. Behav. Brain Res..

[37] Munshi S (2020). Repeated stress induces a pro-inflammatory state, increases amygdala neuronal and microglial activation, and causes anxiety in adult male rats. Brain. Behav. Immun..

[38] Levkovitz Y, Fenchel D, Kaplan Z, Zohar J, Cohen H (2015). Early post-stressor intervention with minocycline, a second-generation tetracycline, attenuates post-traumatic stress response in an animal model of PTSD. Eur. Neuropsychopharmacol..

[39] Liu HY (2018). Chronic minocycline treatment reduces the anxiety-like behaviors induced by repeated restraint stress through modulating neuroinflammation. Brain Res. Bull..

[40] Zhang C, Kalueff AV, Song C (2019). Minocycline ameliorates anxiety-related self-grooming behaviors and alters hippocampal neuroinflammation, GABA and serum cholesterol levels in female Sprague-Dawley rats subjected to chronic unpredictable mild stress. Behav. Brain Res..

[41] Kromer SA (2005). Identification of glyoxalase-I as a protein marker in a mouse model of extremes in trait anxiety. J. Neurosci..

[42] Sartori SB, Landgraf R, Singewald N (2011). The clinical implications of mouse models of enhanced anxiety. Future Neurol..

[43] Tovote P, Fadok JP, Luthi A (2015). Neuronal circuits for fear and anxiety. Nat. Rev. Neurosci..

[44] Dine J (2015). Intranasally applied neuropeptide S shifts a high-anxiety electrophysiological endophenotype in the ventral hippocampus towards a “normal”-anxiety one. PLoS ONE.

[45] Salome N (2004). Neurobiological correlates of high (HAB) versus low anxiety-related behavior (LAB): differential Fos expression in HAB and LAB rats. Biol. psychiatry.

[46] Kraynak TE, Marsland AL, Wager TD, Gianaros PJ (2018). Functional neuroanatomy of peripheral inflammatory physiology: a meta-analysis of human neuroimaging studies. Neurosci. Biobehav. Rev..

[47] Sah A (2012). Anxiety- rather than depression-like behavior is associated with adult neurogenesis in a female mouse model of higher trait anxiety- and comorbid depression-like behavior. Transl. Psychiatry.

[48] Sartori SB, Whittle N, Hetzenauer A, Singewald N (2012). Magnesium deficiency induces anxiety and HPA axis dysregulation: modulation by therapeutic drug treatment. Neuropharmacology.

[49] Echevarria FD, Formichella CR, Sappington RM (2017). Interleukin-6 deficiency attenuates retinal ganglion cell axonopathy and glaucoma-related vision Loss. Front. Neurosci..

[50] Bennett ML (2016). New tools for studying microglia in the mouse and human CNS. Proc. Natl Acad. Sci. USA.

[51] Song L, Lee C, Schindler C (2011). Deletion of the murine scavenger receptor CD68. J. Lipid Res..

[52] Kurushima H (2000). Surface expression and rapid internalization of macrosialin (mouse CD68) on elicited mouse peritoneal macrophages. J. Leukoc. Biol..

[53] Sah A (2019). Epigenetic mechanisms within the cingulate cortex regulate innate anxiety-like behavior. Int. J. Neuropsychopharmacol..

[54] Albrechet-Souza L, Cristina de Carvalho M, Rodrigues Franci C, Brandao ML (2007). Increases in plasma corticosterone and stretched-attend postures in rats naive and previously exposed to the elevated plus-maze are sensitive to the anxiolytic-like effects of midazolam. Hormones Behav..

[55] Moller T (2016). Critical data-based re-evaluation of minocycline as a putative specific microglia inhibitor. Glia.

[56] Lee JS (2019). Antidepressant-like activity of myelophil via attenuation of microglial-mediated neuroinflammation in mice undergoing unpredictable chronic mild stress. Front Pharm..

[57] Wang YL (2018). Microglial activation mediates chronic mild stress-induced depressive- and anxiety-like behavior in adult rats. J. Neuroinflammation.

[58] Li Z, Ma L, Kulesskaya N, Voikar V, Tian L (2014). Microglia are polarized to M1 type in high-anxiety inbred mice in response to lipopolysaccharide challenge. Brain, Behav., Immun..

[59] Anacker C, Hen R (2017). Adult hippocampal neurogenesis and cognitive flexibility—linking memory and mood. Nat. Rev. Neurosci..

[60] Wang CY, Cheng CW, Wang WH, Chen PS, Tzeng SF (2016). Postnatal stress induced by injection with valproate leads to developing emotional disorders along with molecular and cellular changes in the hippocampus and amygdala. Mol. Neurobiol..

[61] Cunningham CL, Martinez-Cerdeno V, Noctor SC (2013). Microglia regulate the number of neural precursor cells in the developing cerebral cortex. J. Neurosci..

[62] Schmidtner AK (2019). Minocycline alters behavior, microglia and the gut microbiome in a trait-anxiety-dependent manner. Transl. Psychiatry.

[63] Schmuckermair C (2013). Behavioral and neurobiological effects of deep brain stimulation in a mouse model of high anxiety- and depression-like behavior. Neuropsychopharmacology.

[64] Muigg P (2007). Altered brain activation pattern associated with drug-induced attenuation of enhanced depression-like behavior in rats bred for high anxiety. Biol. Psychiatry.

[65] Husain MI (2017). Minocycline as an adjunct for treatment-resistant depressive symptoms: a pilot randomised placebo-controlled trial. J. Psychopharmacol. (Oxf., Engl.).

[66] Rodriguez CI (2010). Minocycline augmentation of pharmacotherapy in obsessive-compulsive disorder: an open-label trial. J. Clin. Psychiatry.

[67] Kelly DL (2015). Adjunctive minocycline in clozapine-treated schizophrenia patients with persistent symptoms. J. Clin. Psychopharmacol..

[68] Leigh MJ (2013). A randomized double-blind, placebo-controlled trial of minocycline in children and adolescents with fragile x syndrome. J. Dev. Behav. Pediatr..

[69] Husain MI (2020). Minocycline as adjunctive treatment for treatment-resistant depression: study protocol for a double blind, placebo-controlled, randomized trial (MINDEP2). BMC Psychiatry.

[70] Suzuki S (2016). The infralimbic and prelimbic medial prefrontal cortices have differential functions in the expression of anxiety-like behaviors in mice. Behav. Brain Res..

[71] Kopp BL, Wick D, Herman JP (2013). Differential effects of homotypic vs. heterotypic chronic stress regimens on microglial activation in the prefrontal cortex. Physiol. Behav..

[72] Nozaki K (2020). Antidepressant effect of the translocator protein antagonist ONO-2952 on mouse behaviors under chronic social defeat stress. Neuropharmacology.

[73] Raison CL (2013). A randomized controlled trial of the tumor necrosis factor antagonist infliximab for treatment-resistant depression: the role of baseline inflammatory biomarkers. JAMA Psychiatry.

