# Supplementary material for: Neuroinflammatory alterations in trait anxiety: modulatory effects of minocycline
Source: Transl Psychiatry. 2020 Jul 30;10:256. doi: 10.1038/s41398-020-00942-y (PMC7393101; doi:10.1038/s41398-020-00942-y)
Supplement: Supplementary file 1 — Supplementary Materials and Methods [file 41398_2020_942_MOESM1_ESM.pdf]

Supplementary Materials and Methods

Animals

Male HAB and NAB mice were selectively inbred for their specific anxiety-related behavior at the Department of Pharmacology, Innsbruck Medical University, Innsbruck (Austria), as described previously [47]. HABs and NABs (11-22w) had access to food pellets and water *ad libitum*, and were group-housed in individually ventilated cages under standard laboratory conditions (12:12 light/dark cycle with lights on at 07:00h, 22 ± 2°C, 45-60% humidity). Sample size was chosen using G-power statistics based on an alpha of 0.05 and a beta of 0.02 (statistical power of 80%). The numbers were further validated by the statistics in our previously published studies (Sah et al, 2019) and also of our unpublished observations to estimate that the numbers of animals used in each group were sufficient to arrive to a conclusive result. All experiments were approved by the Austrian Animal Experimentation Ethics Board (Bundesministerium für Wissenschaft Forschung und Wirtschaft, Kommission für Tierversuchsangelegenheiten) and were in compliance with international laws and policies.

Behavioral Testing

All behavioural tests were performed in accordance with established laboratory experimental protocols (44, 69). Behavioral analyses was carried out automatically with VideoMot software tracking (VideoMot 2 Analysis V5.72, TSE) or Ethovision software (12 XT version). The behavioral tests were repeated to replicate the findings.

Elevated Plus Maze

Mice were placed into a plus maze elevated 72cm from the ground, consisting of two closed arms (30 x 5cm) illuminated with red light, and two open arms (30 x 5cm) illuminated with white light at 100 lux, as previously described (37). The test started by placing the mouse on the center platform (5cm x 5cm) facing a closed arm. The behavioural parameters measured included percentage of time spent in the open arms, entries to the open arms, and total distance travelled during the 5min of test duration.

Open Field Test

The open field test consisted of an open box (41 x 41 x 41cm) with a center compartment illuminated at 150lx. Mice were placed in a corner of the open field arena and allowed to explore for 10 min. Parameters measured included total distance travelled, time spent in the center, entries to the center and latency to first entry to center.

Light/Dark Test

The light/dark test was performed in the open field box, but divided into a light arena illuminated at 300lx, and a closed dark chamber at <20lx (black box one third the size of the whole arena) that was accessible through a small door (assigned as transition zone). In brief, mice were placed at the door of the dark chamber and, when it entered the dark chamber, it was allowed to explore the full arena for 10min. Parameters measured included time spent in, number of entries to, distance travelled in, and latency of first entry to the light arena.

Minocycline Treatment

Minocycline dosages for systemic and local administration were chosen according to previous studies showing effects on microglia and behavior (23, 28). Minocycline (Sigma-Aldrich) was dissolved in tap water and was kept in light-protected drinking bottles for oral administration. Each mouse received an average minocycline dosage of 40mg/kg/day by adjusting the concentrations in the bottles according to the mean bodyweight and daily drinking volume per cage, twice a week for 28d. A randomized block design was used in assigning groups to minocycline/tap water treatment.

For local microinjections, minocycline was daily prepared by dissolving in 0.9% sterile saline at a low heat (20µg/µl) and infused bilaterally into the DG once daily, for 5d (short-term) or 11d (long-term). A randomized block design was used for assigning groups to minocycline/saline treatment both, in short-term and long-term treatments.

Stereotaxic Surgery and Microinjections

HAB mice were placed in a stereotaxic frame (David Kopf Instruments) under 2% isoflurane anesthesia. Guide cannulas (25 gauge, 8mm in length) were implanted 1 mm above the left and right DG (AP:-2.18mm, ML: ± 1.40mm, DV: -1.2mm from Bregma) and fixed to the skull with two screws and dental cement. The guide cannulas were closed with dummy stylets in order to prevent clogging. Following surgery, mice were single-housed and received buprenorphine (0.5 mg/kg s.c.) and meloxicam (0.5 mg/kg p.o. via drinking water) for analgesic care for up to 3d. Mice were allowed to recover for at least 7d while being habituated to the experimenter and experimental procedure. Minocycline or saline was locally administered once daily for 5d or 11d. Injection cannulas (31 gauge) were connected to

Hamilton syringes and, following removal of stylets, inserted into the guide cannulas, extending into the DG by an extra 1mm. Either saline or minocycline solution (0.25µl/hemisphere) were infused bilaterally at a speed of 0.1 µl/min. Microcannula were left in place for an additional 2min. Histological verification of probe placement was performed, and only animals with correct probe placement and no visible brain damage were included in the final analysis.

Immunohistochemistry

At 2h following the light/dark test, all mice were terminally anesthetized with an overdose of thiopental (40mg/kg i.p.). Animals were transcardially perfused with 0.9% saline followed by 4% paraformaldehyde in phosphate buffer solution (pH 7.4), as previously described (41). Brains were dissected out and temporarily fixed in 4% paraformaldehyde solution for 2h, followed by permanent storage in phosphate buffer. Coronal sections (40µm) were cut using a vibratome (Leica VT 1000S). Fluorescent immunohistochemistry was performed on free-floating sections, which were incubated overnight at room temperature with the following primary antibodies, using a standard immunohistochemistry protocol (41): goat anti-Iba1 (1:500 Abcam, #ab107159) and rabbit anti-CD68 (1:300 Abcam, #ab125212) or rabbit anti-TMEM119 (1:300 Abcam, # ab209064). This was followed by incubation with secondary donkey anti-goat Alexa Fluor® 647 (1:1000 Jackson Immuno Research) and donkey anti-rabbit Alexa Fluor® 568 (1:1000 Invitrogen) antibodies. The sections were then washed in phosphate buffered saline and mounted onto microscope glass slides (Superfrost Plus, Thermo Scientific), and cover-slipped with Prolong™ Gold Antifade Mountant with 4',6-diamidino-2-phenylindole (DAPI; Invitrogen).

Immunofluorescence Microscopy

In one section per mouse, images of both the left and right DG of the hippocampus (Interaural 1.98mm, Bregma -1.82mm), representative area consisting of polymorphic, hilus and granular cell layers, were taken using a fluorescent microscope (Olympus, Austria), applying a 4x objective to locate specific brain structures and a 20x objective for quantitative analyses. Additional images were taken of the basolateral amygdala (BLA), nucleus accumbens (NAcc), medial prefrontal cortex (mPFC), cingulate cortex and paraventricular nucleus (PVN) of the hypothalamus. For oral minocycline experiment, both the left and right DG (-1.82mm from Bregma) was assessed. For intra-DG minocycline experiment, Iba-1+ cells were counted at the level of (-2.80mm from Bregma) due to significant tissue tear at the level of -1.82mm that was observed in almost all animals. Quantification of immunopositive-cells was assisted by an image analysis system (cellSens Dimension; Olympus).

In addition, Image J software (NIH, USA) was used to objectively quantify Iba1+ cell count (density/mm^2), average cell area size (μm^2), and % Iba1 coverage of the analyzed area. The threshold was selected and validated by comparing automatic and manual counts. An Iba1-positive cell was considered CD68-positive or TMEM119-positive when Iba1 was spatially co-expressed with a positive CD68 or TMEM119 stain respectively, and were manually quantified in equal representative areas.

Experimenters were blinded to animal number codes and thus group allocations, during each behavioral test, microscopic imaging and analysis sessions.

Statistical Analysis

Data analyses were performed with GraphPad Prism 8.0 software (GraphPad Software Inc., USA), following the exclusion of outliers identified by Grubb's test. Anderson-Darling and D'Agostino-Pearson tests, GraphPad Prism 8.0 was used to assess normal distribution. For comparison of two groups the unpaired, two-tailed Student's t test (; GraphPad Prism 8.0) was carried out. There were no comparisons of more than two groups, thus ANOVA analysis is not reported in the manuscript. Majority of the data (51x data sets) passed the equal variance test (F test). For 4x data sets, the variance significantly differed and Welch's correction was added to the Student's t test. Correlational analysis was evaluated by Pearson's co-efficiency analysis, Graphpad prism 8.0. Significance was set at $p<0.05$, and data are presented as means ± standard error of the mean (S.E.M.).

Supplementary Figure and Table Legends

Figure S1. Microglia alterations in select brain regions of HAB vs. NAB mice.

Bars in graphs represent difference between HAB and NAB group means, of Iba1$^+$ microglia density, Iba1$^+$ microglia cell area average, and percentage of brain region covered by Iba1$^+$ staining, in various brain regions. Data are presented as HAB mean minus NAB mean. n=7-11, $^{\#}$p=0.10, *p<0.05**p<0.01***p<0.001 (Student's *t*-test).

Figure S2. Microglial alterations in the ventral dentate gyrus (DG) and medial prefrontal cortex (PFC) of HAB mice. Hyperanxious HAB mice show significantly enhanced Iba1$^+$ cell density in ventral dentate gyrus (DG), and increased Iba1$^+$ cell area average and percentage of

coverage by Iba1+ cells of the ventral DG and PFC, compared to NAB controls. Data are presented as mean ± SEM. n=7-11, *p<0.05**p<0.01 (Student's *t*-test).

Figure S3: Representative image of TMEM119$^+$ (green) and Iba1$^+$ (red) cells in the DG of HAB mice. Iba1 (red) and TMEM119 (green) staining in the dorsal dentate gyrus, outline is DAPI; scale bar 50µm. White arrows are Iba1+TMEM119+ cells, black arrows are Iba1+TMEM119- cells.

Figure S4. Minocycline did not acutely alter anxiety-like behavior in the open field test following the first intra-DG microinjection. On day 1 of 5, no statistically significant differences were found between HAB groups in the open field test (**a**). On day 1 of 11, no statistically significant differences were found between HAB groups in the open field test (**b**). Data are presented as mean ± SEM. n=7-11, *p<0.05 (Student's *t*-test).

Figure S5. Longer period of minocycline induced alterations in the elevated plus maze following the last intra-DG microinjection. On day 11 of 11, intra-DG minocycline HAB group showed increased % of entries to open arm (OA) of the EPM, compared to intra-DG saline HAB group. No statistically significant differences were found between groups for % time spent in OA and total distance travelled. Data are presented as mean ± SEM. n=7-8, *p<0.05 (Student's *t*-test).
