# Supplementary figures and images for: Neuroinflammatory alterations in trait anxiety: modulatory effects of minocycline
Source: Transl Psychiatry. 2020 Jul 30;10:256. doi: 10.1038/s41398-020-00942-y (PMC7393101; doi:10.1038/s41398-020-00942-y)

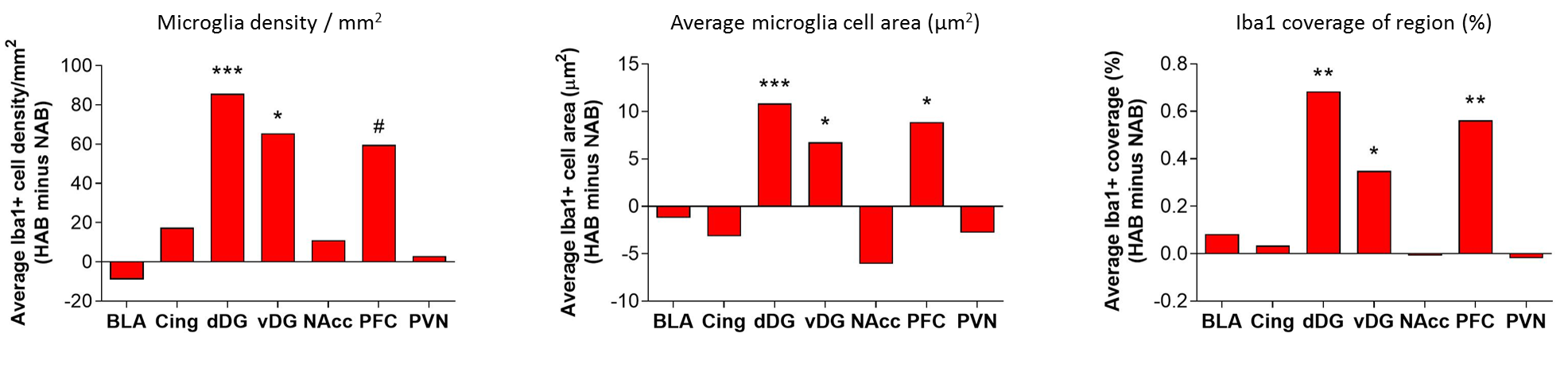

Supplement: Supplementary file 2 — Supplementary Fig S1 [file 41398_2020_942_MOESM2_ESM.png]

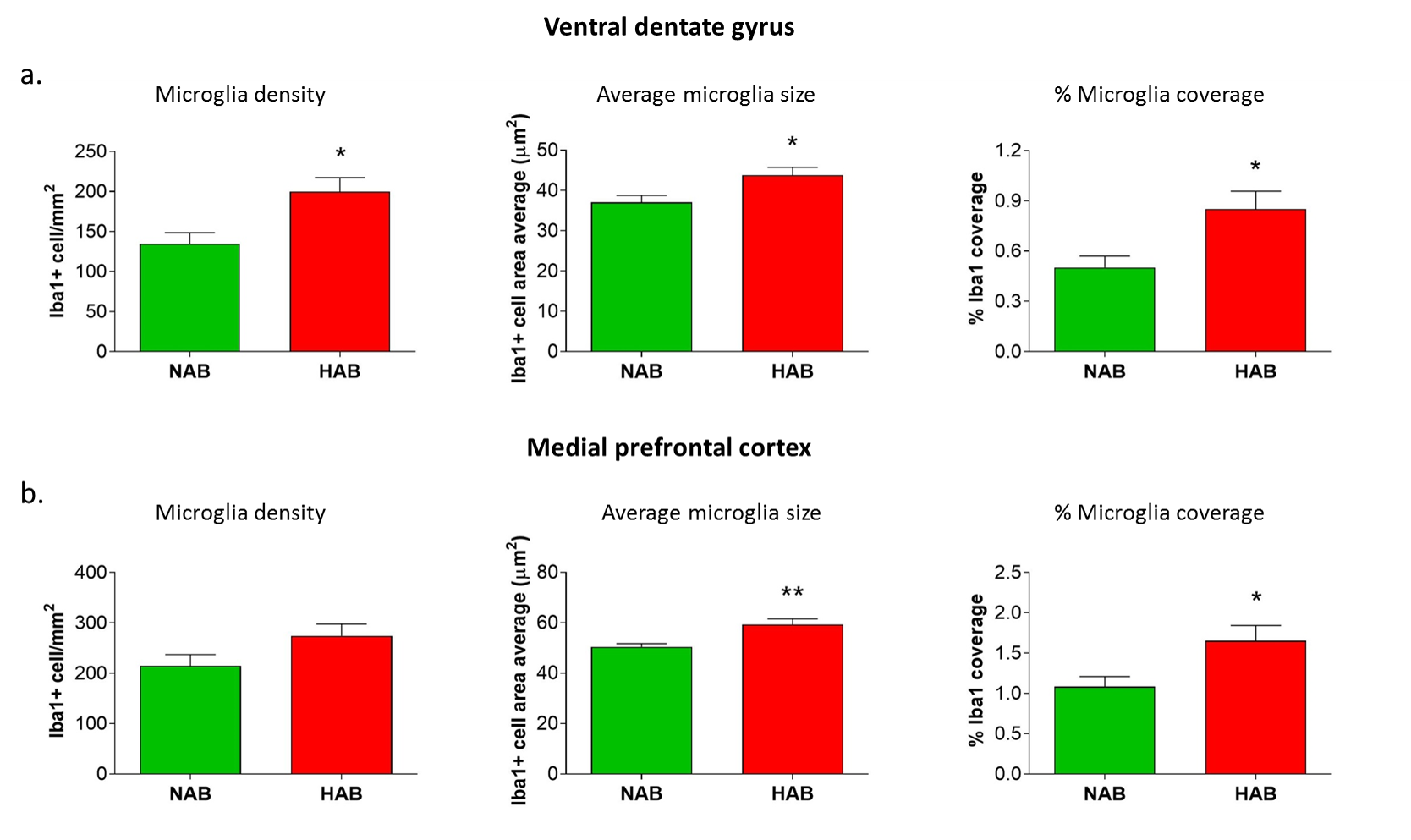

Supplement: Supplementary file 3 — Supplementary Fig S2 [file 41398_2020_942_MOESM3_ESM.png]

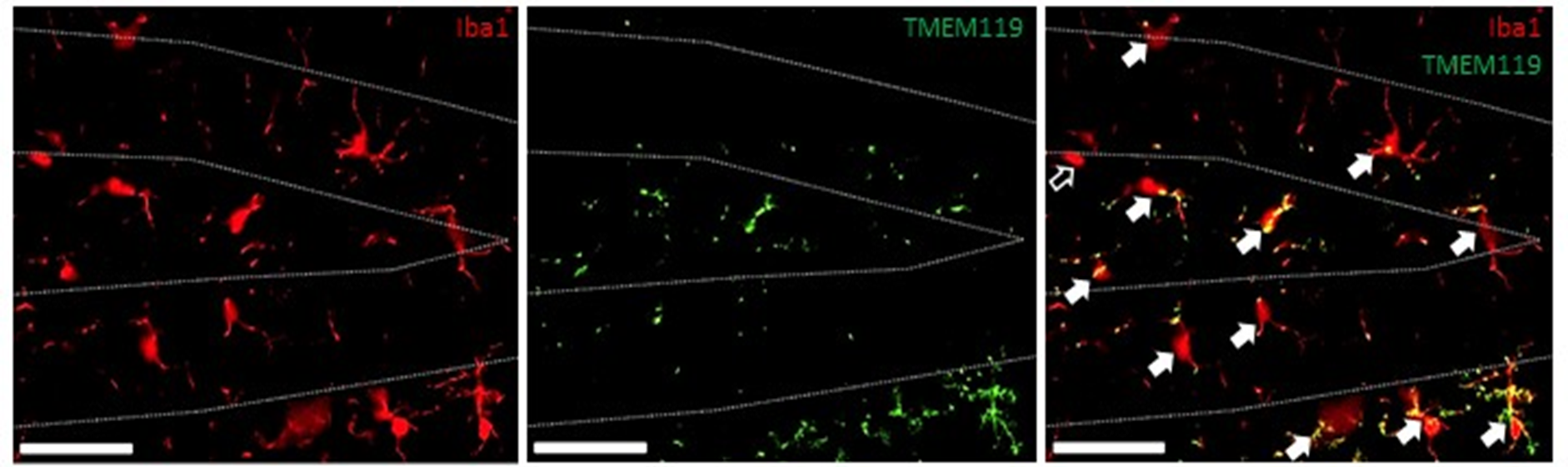

Supplement: Supplementary file 4 — Supplementary Fig S3 [file 41398_2020_942_MOESM4_ESM.png]

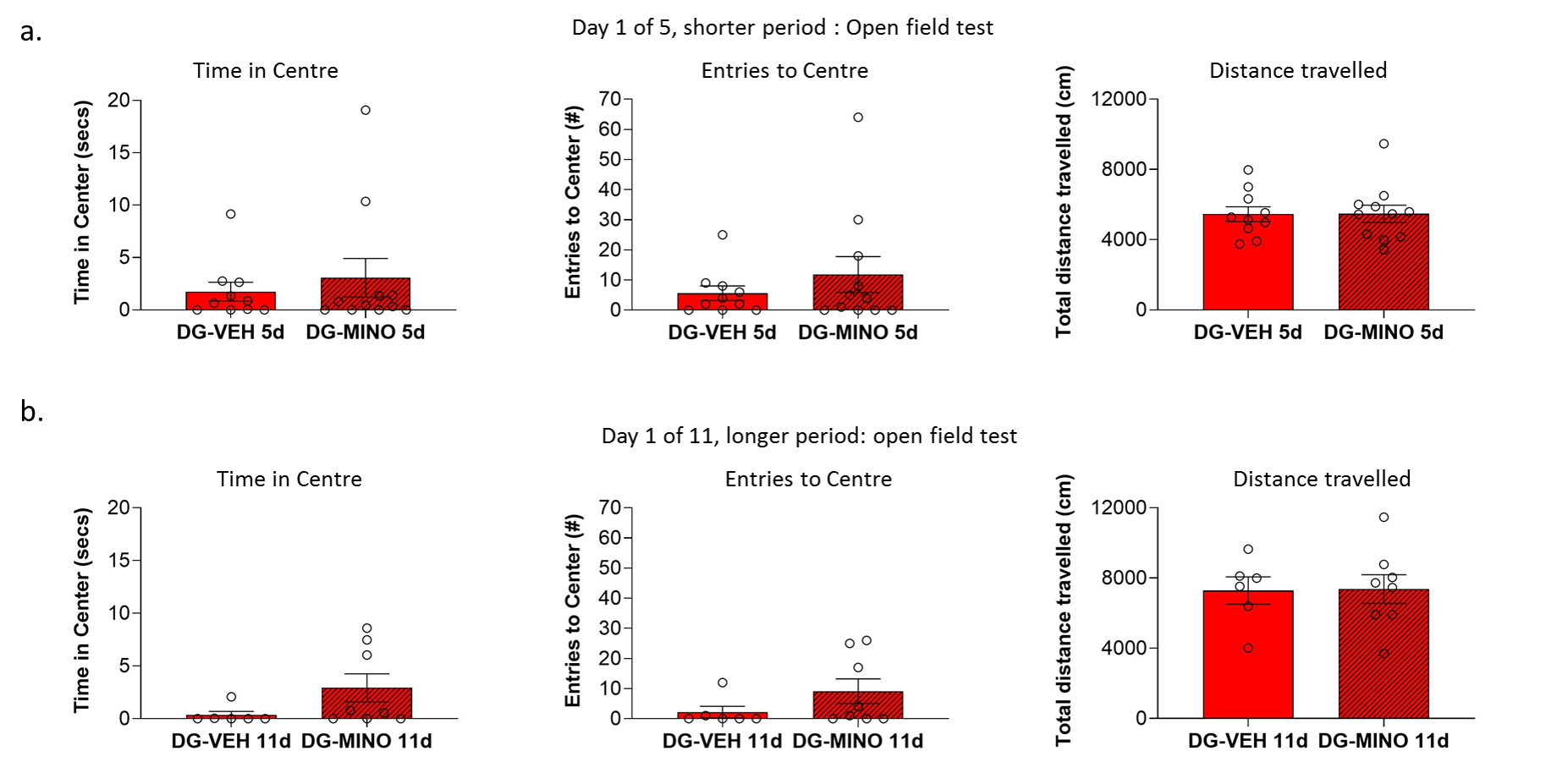

Supplement: Supplementary file 5 — Supplementary Fig S4 [file 41398_2020_942_MOESM5_ESM.png]

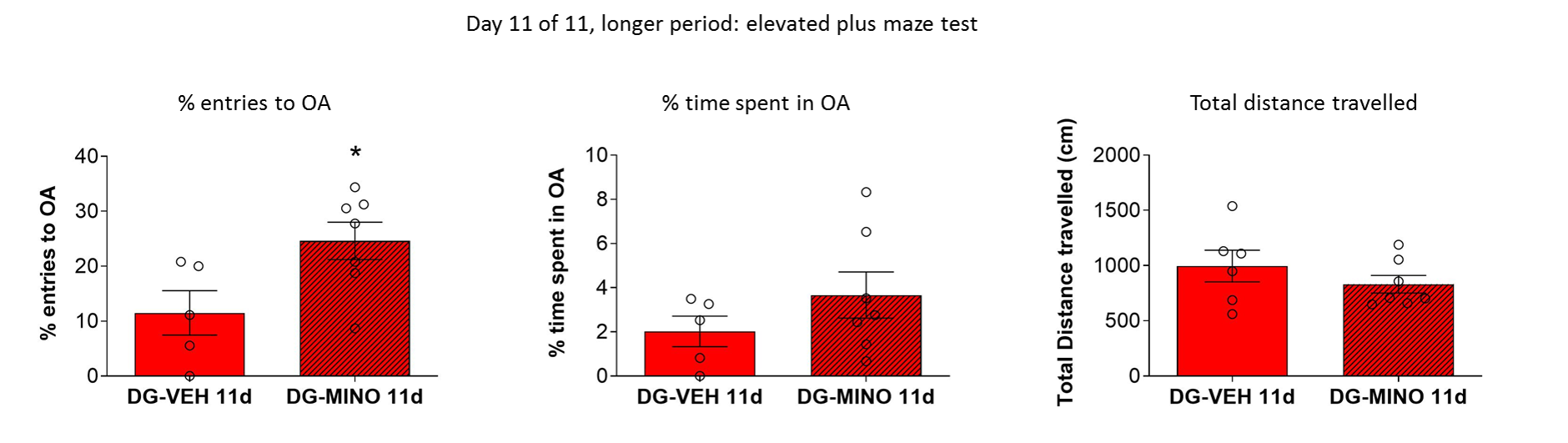

Supplement: Supplementary file 6 — Supplementary Fig S5 [file 41398_2020_942_MOESM6_ESM.png]
